# An empirical examination of the factor structure of compassion

**DOI:** 10.1371/journal.pone.0172471

**Published:** 2017-02-17

**Authors:** Jenny Gu, Kate Cavanagh, Ruth Baer, Clara Strauss

**Affiliations:** 1 School of Psychology, University of Sussex, Falmer, East Sussex, United Kingdom; 2 Department of Psychology, University of Kentucky, Lexington, Kentucky, United States of America; 3 Department of Psychiatry, University of Oxford, Warneford Hospital, Headington, Oxford, United Kingdom; 4 Sussex Partnership NHS Foundation Trust, Hove, United Kingdom; TNO, NETHERLANDS

## Abstract

Compassion has long been regarded as a core part of our humanity by contemplative traditions, and in recent years, it has received growing research interest. Following a recent review of existing conceptualisations, compassion has been defined as consisting of the following five elements: 1) recognising suffering, 2) understanding the universality of suffering in human experience, 3) feeling moved by the person suffering and emotionally connecting with their distress, 4) tolerating uncomfortable feelings aroused (e.g., fear, distress) so that we remain open to and accepting of the person suffering, and 5) acting or being motivated to act to alleviate suffering. As a prerequisite to developing a high quality compassion measure and furthering research in this field, the current study empirically investigated the factor structure of the five-element definition using a combination of existing and newly generated self-report items. This study consisted of three stages: a systematic consultation with experts to review items from existing self-report measures of compassion and generate additional items (Stage 1), exploratory factor analysis of items gathered from Stage 1 to identify the underlying structure of compassion (Stage 2), and confirmatory factor analysis to validate the identified factor structure (Stage 3). Findings showed preliminary empirical support for a five-factor structure of compassion consistent with the five-element definition. However, findings indicated that the ‘tolerating’ factor may be problematic and not a core aspect of compassion. This possibility requires further empirical testing. Limitations with items from included measures lead us to recommend against using these items collectively to assess compassion. Instead, we call for the development of a new self-report measure of compassion, using the five-element definition to guide item generation. We recommend including newly generated ‘tolerating’ items in the initial item pool, to determine whether or not factor-level issues are resolved once item-level issues are addressed.

## Introduction

Until recently, the scientific study of compassion towards others has been hampered by a lack of definitional and measurement clarity [1]. This contrasts to the long-standing emphasis on compassion as a fundamental part of our humanity in Eastern contemplative traditions, such as Buddhism, and major world religions [2, 3]. In Buddhism, compassion, conceptualised as the “heart that trembles in the face of suffering” [4], is regarded as essential to gaining wisdom [5], happiness [6], and freeing our minds from destructive emotions [7]. In support of this, emerging research has linked compassion to a number of positive constructs, such as increased happiness and self-esteem [8], increased social connectedness, greater wellbeing, and lowered levels of loneliness [9]. Additionally, the 2015 *World Happiness Report* [10] highlighted compassion-related constructs, altruism and prosocial behaviour, as one of the four constituents of wellbeing.

Over the past few decades, scientific and societal interest in compassion has blossomed in many different sectors. There is a growing awareness of the importance of placing compassion at the heart of healthcare for the benefit of patients, staff, and healthcare organisations. In the US, compassion is integral to the Institute of Medicine’s definition of patient-centred care [11] and according to a US survey, 81% of 800 service users and 71% of 510 physicians agreed that compassionate care has an impact on whether a patient lives or dies [12]. Similarly, compassion is considered to be one of the six fundamental values in the UK National Health Service constitution [13]. The growing awareness of the importance of compassion is connected to concerns about compassion wearing thin in cases of work-related burnout [14], a common problem in the emotionally demanding healthcare profession. The benefits of compassion have also been recognised in the education sector, with organisations such as ‘Mind with Heart’ (www.mindwithheart.org), the ‘Compassion in Education’ foundation (www.coedfoundation.org.uk), and the ‘Resilience, Wellbeing, Success’ programme (www.rws.today) dedicated to equipping teachers and learners with the skills necessary to promote more compassionate learning environments.

Considering compassion as an innate capacity is also gaining traction in scientific circles. From an evolutionary perspective, compassion is thought to confer reproductive advantages through its role in the care-giving system for protecting and nurturing vulnerable offspring (e.g., [15, 16]). In humans and other higher-order primates, as the mind increased in complexity and competency, compassion towards offspring was thought to generalise to others in need (e.g., [15, 16]), partly because it enables advantageous cooperative relationships with non-kin (e.g., [17]). These reproductive advantages mean that compassion is a desirable criterion in mate selection (e.g., [17]). This evolutionary perspective echoes that of Darwin [18], who considered compassion, which he termed ‘sympathy’, to be an instinct which confers survival advantages, noting that: “Sympathy will have been increased through natural selection; for those communities, which included the greatest number of the most sympathetic members would flourish best, and rear the greatest number of offspring” (p. 130). Interestingly, recent studies also show that people regard moral capacities (including compassion) as the mental faculty most essential to their sense of self and how they perceive the identity of others, over and above autobiographical memories, personality, and desires and preferences [19].

### Cultivating compassion

Several interventions have the potential to enhance compassion (or outcomes related to compassion). Programs developed to explicitly target the cultivation of compassion include Compassion Cultivation Training [20], Cognitively-Based Compassion Training (designed by Lobsang Tenzin Negi), Compassionate Mind Training [21], Mindfulness-Based Compassionate Living [22], Mindful Self-Compassion [23], and other compassion-based contemplative practices such as Loving Kindness Meditation. In addition, mindfulness-based interventions such as mindfulness-based cognitive therapy [24, 25] and mindfulness-based stress reduction [26] are examples of interventions which may implicitly raise participants’ level of compassion. A number of randomised controlled trials have found these interventions to be effective for improving a broad range of psychological outcomes (e.g., [27, 28, 29, 30, 31, 32, 33]). However, none of these studies measured compassion directly; improvements in compassion were inferred from increases in constructs thought to be related to compassion. This is a significant limitation–we do not know if enhanced compassion is the mechanism through which these interventions are having their beneficial effects. The evidence for compassion-based interventions would be considerably strengthened by measuring compassion itself; however, this has been impeded by a lack of definitional clarity.

### Conceptualising compassion

Despite the growing interest in compassion towards others, and the development of interventions designed to cultivate compassion, scientific progress has been hindered by the number of different ways in which the construct has been conceptualised. This is further complicated by the existence of closely-related constructs such as empathy, sympathy, love, altruism, kindness, and pity, and the tendency to use these terms interchangeably. In their comprehensive review of empirical studies of compassion towards others, Goetz, Keltner, and Simon-Thomas [34] found support for compassion as a unique, multidimensional construct which can be differentiated from related states in terms of the appraisal processes, affective experience, physiological responses, and patterns of behaviour involved when encountering others in need. Their findings support the evolutionary account and further justify the study of compassion as a construct in its own right. Neuroscientific findings of differential activation of brain regions in response to empathy and compassion training [35] lend additional support to the perspective of compassion as a distinct construct.

A closely-related construct which has received more empirical attention is compassion directed towards the self, or self-compassion [36]. The Buddhist perspective, which has a more nuanced view of the duality of self and others, regards the underlying processes of compassion as common to our experience regardless of the object of compassion (self or others). Moreover, self-compassion is seen as supportive of compassion towards others [37]. However, current research indicates that the relationship between compassion towards others and self-compassion is not straightforward. Neff and Pommier [38] have failed to find a relationship between self-compassion and other-focused constructs (other-compassion, compassion for humanity, empathic concern, and altruism) in a student sample, but found significant associations between self-compassion and other-focused constructs in samples of community adults and meditators. Using an alternative measure of compassion towards others, Pommier [39] also failed to find a significant relationship between this and self-compassion in a student sample. It is currently uncertain whether the lack of association reflects a separation between these two forms of compassion, only occurs in students or in Western cultures, or can be explained by limitations of the measures used in these studies (e.g., [1]).

Strauss and colleagues [1] reviewed the compassion literature and consolidated the range of conceptualisations of compassion into one multifaceted definition. They concluded that compassion entails five elements that apply to the self or others: 1) recognising suffering, 2) understanding the universality of suffering in human experience, 3) feeling for the person suffering and emotionally connecting with their distress, 4) tolerating any uncomfortable feelings aroused (e.g., fear, disgust, distress, anger) so that we remain accepting and open to the person in their suffering, and 5) acting or being motivated to act to alleviate the suffering. Strauss and colleagues [1] also systematically reviewed existing questionnaire measures of compassion and evaluated each measure’s psychometric properties. The authors concluded that none of the scales reviewed comprehensively assessed all elements of compassion and many scales had poor or inadequately tested psychometric properties. They call for the empirical examination of the proposed five elements of compassion and ultimately, the development of a psychometrically-robust measure that comprehensively captures the key elements of compassion. These steps are crucial if we are to progress the various strands of compassion research discussed, including investigating how compassion relates to other constructs and evaluating of the effectiveness of interventions to cultivate compassion.

### The current study

No studies have empirically investigated the five-element definition of compassion proposed by Strauss et al. [1]. This is an essential first step to deepening our understanding of compassion, developing a high quality measure, and furthering research in this field. The current study used factor analyses, relying primarily on existing self-report measures of compassion, to determine whether items from this combined pool could be identified that provide preliminary support for the proposed five-element definition. However, previous examination of these items [1] suggested two potential problems. First, the combined pool might contain too few items to represent some of the five elements (e.g., tolerating uncomfortable feelings, understanding the universality of suffering). Second, some of the existing items may have poor face validity, as they appear to reflect related constructs, such as empathy and altruism. Therefore, sufficient new items were generated to provide a meaningful test of the proposed five-factor structure. The study consisted of three stages.

Stage 1 involved a systematic consultation with expert groups to review items from existing self-report measures of compassion and to generate additional items. Strauss et al.’s [1] comprehensive five-element definition of compassion was used to guide the item generation and review process. Stage 1 resulted in a pool of 80 items for factor analysis: 54 items from existing compassion questionnaires and 26 items generated by expert consultants (see later for details).

Stage 2 used exploratory factor analysis (EFA) in a sample of University students to examine the factor structure of the pool of compassion items generated in Stage 1. Although Strauss et al. [1] proposed a five-element structure of compassion based on their review of the theoretical literature, EFA was conducted to remove redundant items and to provide an empirical examination of the structure of the item pool without assuming that the five-element model would emerge. Using EFA therefore allows us to explore the factor structure of compassion naturally emerging from the data without privileging any theorised definition. Stage 3 aimed to validate the factor structure found in Stage 2 in an independent student sample using confirmatory factor analysis (CFA). This study received ethical approval from the University of Sussex Sciences and Technology Cross-Schools Research Ethics Committee.

## Stage 1: Item generation and review

### Method

#### Participants

Three groups of experts were consulted to review and contribute to the items from existing self-report measures of compassion: 1) experts in teaching contemplative approaches to others (mindfulness or compassion-based interventions), 2) experts in delivering care to others in healthcare or other pastoral care settings (e.g., healthcare staff, teachers, university lecturers), and 3) experts by experience (e.g., recipients of healthcare). Experts were recruited through e-mail invitations to an established mental health service user research group, the UK Network for Mindfulness-based Teacher Training Organisations, and faculty at the School of Psychology at the host University. Fifteen experts (66.67% female, 86.67% Caucasian) completed the consultation: eight experts in teaching contemplative approaches to others, five experts in delivering care to others in healthcare or other pastoral care settings, and two recipients of healthcare. Experts’ ages ranged from 35 to 77 years (*M* = 52.80, *SD* = 11.52).

#### Compassion measures

Although nine compassion measures were identified in Strauss et al.’s [1] review, only items from the following four measures were included in this consultation: the compassionate love scale (CLS) [40], Pommier’s [39] compassion scale (CS-P), relational compassion scale (RCS) [41], and Martins et al.’s [42] compassion scale (CS-M). Excluded from the consultation were the Santa Clara brief compassion scale (SCBCS) [43], compassionate care assessment tool (CCAT) [44], Schwartz Center compassionate care scale (SCCCS) [45], self-compassion scale (SCS) [36], and short form of the self-compassion scale (SCS-SF) [46].

The SCBCS was not included because it is a shortened version of the CLS. Items from the CCAT and the SCCCS were not included because they measure patients’ ratings of compassionate care received from healthcare staff, and were not self-report measures of compassion. The SCS and SCS-SF were not included for several reasons. First, although compassion towards others and self-compassion have been theorized to be part of the same overarching construct, research findings have generally not supported a relationship between these two constructs. Second, items from the SCS and SCS-SF could not be easily reworded to apply to compassion towards others, or compassion more generally. Lastly, the CS-P, which was included in this study, was developed based on the factor structure of the SCS and included the same elements (kindness, mindfulness, common humanity). Therefore, although items in the CS-P and SCS are not the same, the way in which these measures conceptualise the key elements underpinning compassion towards others and self-compassion is the same.

Compassionate love scale [40]. The 21-item CLS measures compassionate or altruistic love for close others and all of humankind, including strangers. Strauss et al. [1] noted that the CLS includes items related to four of the five elements in their definition: emotionally connecting with other people’s suffering, understanding something about their experience/suffering as a fellow human being, accepting and not judging them (implying tolerance), and being motivated to help them.

Compassion scale [39]. The 24-item CS-P mirrors the factor structure of Neff’s [36] SCS and consists of the same three subscales: kindness versus indifference, common humanity versus separation, and mindfulness versus disengagement. The CS-P contain items which capture four of the five elements in Strauss et al.’s [1] definition: recognising suffering, emotionally connecting with another person’s distress, understanding their experience as a fellow human being, and being motivated to act or acting to alleviate suffering.

Compassion scale [42]. The 10-item CS-M was developed to tap into five aspects of compassion: generosity, hospitality, objectivity, sensitivity, and tolerance across social networks and relationships. Two versions of each item exist. For the first three questions, one version relates specifically to friends and the other to strangers. For the fourth question, one version relates to friends and the other to family, and for the fifth question, one version relates to the self and the other version to other people. Therefore, this scale consists of five unique items. CS-M items focus exclusively on compassionate acts and therefore capture only the acting to alleviate suffering element of Strauss et al.’s [1] five-element definition.

Relational compassion scale [41]. The 16-item RCS consists of four subscales measuring respondents’ compassion for others, self-compassion, beliefs about how compassionate other people are to each other, and beliefs about how compassionate other people are to them. The four items from the beliefs about how compassionate other people are to each other subscale were not included in the consultation because they do not directly involve the participants themselves. RCS items capture four of the five elements from Strauss et al.’s [1] review: recognising suffering, accepting and not judging others (implying tolerance), emotionally connecting with their distress, and acting to alleviate suffering.

#### Procedure

In order for experts to review the face validity of each item from existing compassion measures and generate additional items (using Strauss et al.’s [1] definition as a guide), prior to consultation, two researchers in contemplative approaches met with the first author and designated each item from the included self-report compassion measures to one of the five elements from Strauss et al.’s [1] definition. Disagreements were resolved through discussion and there was 100% agreement for the final allocations.

Where strictly necessary, the two experts and the first author met together to reword items to overcome the limitations highlighted by Strauss et al. [1] and to fit the format of the question and response scale chosen by the research team (“Thinking about yourself in general, indicate how true the following statements are of you by choosing the appropriate number on a scale from 1 (not at all true of me) to 7 (completely true of me)”). Examples include changing items worded as questions to statements, altering items to refer to all other people (not specifically close others, friends, or strangers), and removing frequency terms (e.g., sometimes, often). However, changes to wording were kept to an absolute minimum; we endeavoured to keep as many items as possible in their original form. A total of 62 items were gathered from existing compassion measures.

The online consultation, implemented on Bristol Online Surveys (BOS; www.onlinesurveys.ac.uk), provided experts with Strauss et al.’s [1] five-element definition and instructed them to: A) decide for each element whether existing items adequately represented this aspect of compassion, by selecting ‘yes’ or ‘no’, and B) suggest up to five additional items for each element if they thought that any were missing, using open text boxes. Experts could also leave general comments about the items designated to each element. It was decided *a priori* that in part A), for each item, at least 50% of experts needed to respond ‘yes’ to demonstrate that the item adequately represented the element; if more than half of experts agreed that an item was not a good indicator of an element, that item would be removed for Stage 2. It was also decided *a priori* that in part B), suggested items would be included if they: 1) did not semantically overlap with an existing item (i.e. where the wording was different but the meaning the same or very similar), and 2) adequately captured the relevant element of compassion. Two members of the research team reviewed each suggested additional item and came to an agreement concerning whether or not items should be included.

### Results

Based on the feedback from experts in part A), eight of the 62 items were removed because they were not deemed to adequately represent aspects of compassion by over 50% of respondents. These were: “I like to listen to other peoples' experiences’” (RCS), “I feel a selfless caring for other people” (CLS), “I feel considerable compassionate love for people around me” (CLS), “I would rather suffer myself than see someone else suffer” (CLS), “I would rather engage in actions that help others than engage in actions that would help me” (CLS), “I would be willing to do the right thing even if it puts others at risk” (CS-M), “I would be willing to allow others pleasure of something even if it caused me pain” (CS-M), and “I don’t think much…” (CS-P; for the full item, refer to [39]). Twenty-six items were added based on experts’ suggestions in part B). Therefore, in total, the pool of compassion items for Stage 2 of this study comprised of 80 items (refer to S1 File for the pool of compassion items).

## Stage 2: Exploratory factor analysis

### Method

#### Participants and procedure

Participants were 206 University students (77.18% female). Their ages ranged from 18 to 50 years (*M* = 22.30, *SD* = 4.67). Inclusion criteria were that participants must either be undergraduate or postgraduate students at the host University. There were no other exclusion criteria. Participants completed a survey containing the 80 compassion items derived from Stage 1 of this study in exchange for course credits or entry into a prize draw. The survey was hosted on BOS and items were arranged such that they alternated among the five elements.

The online survey was part of a larger study and also contained the following self-report measures: the 24-item Five Facet Mindfulness Questionnaire (FFMQ) [47], 12-item Self-Compassion Scale [46], 21-item Depression, Anxiety, and Stress Scale [48], the 7-item Short Warwick-Edinburgh Mental Wellbeing Scale [49], and the 21-item Interpersonal Reactivity Index [50].

#### Sample size

Rules of thumb relating to sample size for EFA are generally regarded as not useful or valid [51]. Instead, studies have shown that adequate sample size should be determined by the nature of the data. MacCallum et al. [51] stress the importance of level of item communality, or the proportion of variance in a variable shared with other variables [52], in determining sample size. With a sample size of 206 participants, the factor structure should be stable provided communalities are around .50 or greater and factors are well-determined (at least three items per factor and strong loadings of items to factors) [51].

#### Statistical analyses

All analyses were conducted using SPSS version 22 [53]. Negatively-phrased items were reverse-coded prior to analysis. Preliminary analyses involved examining the intercorrelation between compassion items to identify and remove variables which did not correlate with any other variables or correlated highly with other variables (*r* > .90). Additionally, Bartlett’s test of sphericity was checked, and sampling adequacy was examined using the Kaiser-Meyer-Olkin (KMO) measure to assess the suitability of the data for EFA. EFA was conducted using principal components analysis (PCA), and oblique rotation (direct oblimin) was selected to allow for correlations among the factors.

Within each identified factor, the highest loading items (with factor loadings of at least .50) were selected to be tested in the CFA in Stage 3. A minimum of three and maximum of five items were selected per factor to ensure that factors were well-determined and of manageable length. Items which were not theoretically related to the highest loading items in a factor were not included.

### Results

The initial PCA yielded 16 factors with eigenvalues greater than 1.0, which collectively accounted for 68.98% of the total variance. However, the scree plot indicated that a five factor solution should be extracted. Costello and Osborne [54] recommend using the scree plot to decide the number of factors to retain, because including all factors with eigenvalues greater than 1.0 is one of the least accurate methods [55]. Therefore, a second PCA was conducted which specified the extraction of five factors.

The five factor solution explained 49.96% of the total variance. There were no variables with problematically low correlations, or correlated highly with other variables (*r* > .90). The overall KMO value was .92, KMO values for individual variables were > .50, and Bartlett’s test of sphericity was significant (*p* < .001). These results indicate that EFA was appropriate for the data. The factor structure and loadings of items to factors are presented in Table 1. The origin of each item is also given (CLS, CS-P, RCS, CS-M, or expert consultation). Only items with strong factor loadings (.50 or greater) and low loadings on all other factors (a difference of at least .20 between the highest loading and loadings on to other factors) are included in Table 1. Most item communalities were greater than .50, which indicates that the sample size was adequate [51].

**Table 1:** Factor structure and loadings of compassion items in a sample of 206 students.

Item source and content	Factor loading ^a^				
	1	2	3	4	5
**Factor 1**: Acting to help/alleviate suffering					
*CLS: If given the opportunity, I am willing to make sacrifices in order to let other people achieve their goals in life.	.73				
*CLS: If a person needs help, I would do almost anything I could to help him or her.	.67				
*CLS: I want to spend time with others so that I can find ways to help enrich their lives.	.65				
*CLS: One of the activities that provides me with the most meaning to my life is helping others.	.65				
*EC: If someone is suffering I go out of my way to help them if I can.	.62				
CLS: When someone is troubled, I feel extreme tenderness and caring.	.59				
CLS: I spend a lot of time concerned about the well-being of other people.	.58				
CLS: When I see people feeling sad, I feel a need to reach out to them.	.57				
RCS: When other people are emotionally upset I treat them with kindness and care.	.52				
**Factor 2**: Tolerating uncomfortable feelings					
*EC: When I see someone feeling upset I feel so overwhelmed by my emotions that I find it difficult to help them. ^R^		.64			
*EC: When someone is suffering it can be hard to help them because it is so upsetting. ^R^		.59			
*EC: I get carried away by my own emotional response to other people's problems or suffering. ^R^		.58			
**Factor 3**: Understanding the universality of suffering					
*CS-P: I believe that suffering is just… ^b^			.77		
*CS-P: Despite my differences with others, I know… ^b^			.68		
*CS-P: It is important to me to recognize that… ^b^			.60		
CS-P: When people tell me about their problems, I… ^b^			.56		
*CS-P: I know that everyone feels down… ^b^			.52		
**Factor 4**: Recognising suffering					
*CS-P: I notice when people are upset… ^b^				-.75	
*EC: I notice when someone is different from how they usually are.				-.74	
*EC: I find it easy to recognise when someone is suffering or in need.				-.67	
*EC: I find it difficult to notice when people are upset. ^R^				-.67	
*EC: I can understand how people are feeling even if I do not identify with their experiences.				-.55	
**Factor 5**: Emotional connection					
*CS-P: When people talk about their problems… ^b^ ^R^					-.76
*CS-P: I feel detached from others… ^b^ ^R^					-.71
CS-P: When others are feeling troubled, I… ^b^ ^R^					-.70
*CS-P: I don't feel emotionally… ^b^ ^R^					-.67
*EC: It is hard for me to relate to others when I see them suffering. ^R^					-.67
CS-P: I tune out when people… ^b^ ^R^					-.66
*CS-P: I don't concern myself… ^b^ ^R^					-.65
CS-P: I try to avoid people who… ^b^ ^R^					-.62
CS-P: I am cold to… ^b^ ^R^					-.59
EC: If someone is in distress or trouble, I wait for other people to respond first. ^R^					-.57
CS-P: I can't really connect with… ^b^ ^R^					-.57
CS-P: When I see someone feeling down, I… ^b^ ^R^					-.56
CS-P: When people cry in front of… ^b^ ^R^					-.55

CLS = Compassionate Love Scale; CS-P = Pommier’s Compassion Scale; EC = expert consultation; RCS = Relational Compassion Scale.

^a^ Items with factor loadings of less than .50 or cross-loadings (a difference of less than .20 between the highest loading and loadings on to other factors) are suppressed.

^b^ Only item stems (50% or less of the full items) are given for items from the CS-P. For full items, please refer to Pommier [39].

^R^ Items are negatively-phrased and have been reverse-coded prior to analysis.

*Item included in the confirmatory factor analysis in Stage 3.

Items in factor 1 generally appear to represent motivation to act or acting to help/alleviate suffering, factor 2 represents tolerating uncomfortable feelings so that we are able to help, factor 3 represents understanding the universality of suffering in human experience, factor 4 represents recognising suffering, and factor 5 represents emotionally connecting with the person in distress. These factors support Strauss et al.’s [1] five-element definition. Asterisks next to items in Table 1 indicate selected items for CFA.

## Stage 3: Confirmatory factor analysis

### Method

#### Participants and procedure

Participants were a new sample of 256 undergraduate and postgraduate students at the host University (81.64% female, 84.38% Caucasian). Their ages ranged from 18 to 50 years (*M* = 19.87, *SD* = 4.08). Participants completed a survey on BOS containing the selected compassion items from Stage 2 of this study in exchange for course credits. The online survey was part of a larger study and also contained the Big Five Inventory measure [56].

#### Statistical analyses

Three CFA models were tested using maximum-likelihood estimation with robust standard errors (MLR) conducted in MPlus version 6. MLR was used because it produces standard errors and a chi-square test statistic that are robust to deviations from normality. First, a single-factor model in which all item are indicators of a single compassion factor was tested. Next, the five-factor model derived from the EFA in Stage 2 was tested. Lastly, a hierarchical model was tested, in which the five factors are components of a broad compassion factor. Six fit indices were used to evaluate the fit of the models to the data: the comparative fit index (CFI) [57], the root mean square error of approximation (RMSEA) [58], the non-normed fit index (NNFI) [59], the standardised root mean square residual (SRMR), the Akaike information criterion (AIC) [60], and the relative chi-square test.

As a rule of thumb, CFI and NNFI should be greater than or close to .95 to indicate acceptable fit [61], an RMSEA value of .05 or less is considered a good fit, .08 indicates acceptable fit, and .10 or more a poor fit [62], and the SRMR should be less than .08 for acceptable fit [61]. Relative chi-square values were obtained by dividing the chi-square test statistic by the degrees of freedom. Good fit is indicated by relative chi-square values of less than or equal to 2 and acceptable fit is indicated by values between 2 and 3 [63]. The significance of the chi-square statistic was not used as a primary measure of model fit because of its hypersensitivity (e.g., to large sample sizes, non-normality, and large correlations between variables) [64]. The AIC was used to compare the fit of the three models, with lower values indicating superior fit. In addition to these fit indices, the significance of factor loadings was also considered when interpreting which model provided a better fit to the data.

### Results

Table 2 shows the fit indices for the three CFA models. Bold indices (CFI, RMSEA, NNFI, SRMR, and relative χ^2^) indicate acceptable fit. All indices show that the fit of the one-factor model to the data was poor, which suggests that items are not direct indicators of an overall compassion factor. RMSEA, SRMR, and relative χ^2^ values for the five-factor model indicated good model-data fit. However, CFI and NNFI values for this model were just under the threshold for acceptable fit. Similarly, for the five-factor hierarchical model, RMSEA, SRMR, and relative χ^2^ values indicated good fit but CFI and NNFI values suggested marginally-acceptable fit. In the five-factor hierarchical model, loadings of all five factors to the overarching compassion factor were significant (Table 3). This suggests that the five factors can be seen as elements of an overall compassion construct. All loadings of items on to relevant factors in both the five-factor and five-factor hierarchical model were also significant. Based on both the fit indices and significance of factor loadings, the five-factor hierarchical model can be interpreted as providing a better fit compared to the other two models.

**Table 2:** Fit indices for the three CFA models tested in a sample of 256 students.

Model	CFI	RMSEA [90% CI]	NNFI	SRMR	Relative χ^2^ (χ^2^ / *df*)	χ^2^ (*df*)	AIC
One-factor	.707	.095 [.087, .103]	.676	.085	3.318	693.384 (209)	16821.585
Five-factor	.937	**.045 [.035, .055]**	.927	**.057**	**1.522**	302.957 (199)	16383.586
Five-factor hierarchical ^a^	.924	**.049 [.039, .059]**	.914	**.063**	**1.616**	329.716 (204)	16403.647

AIC = Akaike information criterion; CFA = confirmatory factor analysis; CFI = comparative fit index; CI = confidence interval; NNFI = non-normed fit index; RMSEA = root mean square error of approximation; SRMR = standardised root mean square residual.

Bold indices (CFI, RMSEA, NNFI, SRMR, and relative χ^2^) indicate acceptable fit when rounded up or down to two decimal places.

^a^ Five-factor hierarchical refers to the model in which all five factors load on to an overarching compassion factor.

**Table 3:** Standardised loadings of factors to an overarching compassion factor in the five-factor hierarchical CFA model (*N* = 256).

Factor	Standardised loading (*SE*)
Recognising suffering	0.77 (0.05)*
Understanding the universality of suffering	0.61 (0.08)*
Emotional connection	0.90 (0.04)*
Tolerating uncomfortable feelings	0.25 (0.10)*
Acting to alleviate suffering	0.82 (0.05)*

*p < .05

Table 4 displays the factor intercorrelations in a five-factor model. All intercorrelations were significant with the exception of the correlation between the ‘tolerating uncomfortable feelings’ and ‘acting to alleviate suffering’ factors.

**Table 4:** Factor intercorrelations in the five-factor model (*N* = 256).

	1	2	3	4	5
1. Recognising suffering	-				
2. Understanding the universality of suffering	.51*	**-**			
3. Emotional connection	.68*	.51*	-		
4. Tolerating uncomfortable feelings	.25*	.29*	.28*	**-**	
5. Acting to alleviate suffering	.63*	.51*	.75*	.01	**-**

*p < .05

## General discussion

This current study used self-report items to empirically examine the five-element definition of compassion proposed by Strauss et al. [1]. The study consisted of three stages: a systematic consultation with expert groups to review items from existing self-report measures of compassion and generate additional items (Stage 1), EFA to identify the factor structure of compassion and remove redundant items (Stage 2), and CFA to validate the factor structure found in Stage 2 (Stage 3).

Findings from the EFA supported a five-factor structure of compassion consistent with Strauss et al.’s [1] five-element definition. The five factors emerging from the analysis were: 1) recognising suffering, 2) understanding the universality of suffering in human experience, 3) emotionally connecting with the person in distress, 4) tolerating uncomfortable feelings so that we are able to help, and 5) being motivated to act or acting to help/alleviate suffering. Findings from the CFA provided promising support for this five-factor model and a five-factor hierarchical model, with all but two indices demonstrating good fit and significant item loadings and loadings of all five factors to an overarching compassion factor.

However, inspection of factor intercorrelations (in a five-factor model) and the magnitude of loadings of factors to an overarching compassion factor (in a five-factor hierarchical model) highlight issues with the ‘tolerating uncomfortable feelings’ factor, which did not correlate significantly with the ‘acting to alleviate suffering’ factor and had the smallest factor loading (standardised loading = .25; standardised loadings of other factors = .61 to .90). The non-significant correlation between tolerating and acting factors was unexpected, given the inclusion of both elements in theoretical conceptualisations of compassion (e.g., [4, 65]), and it is uncertain why tolerating would be significantly related to all factors but the acting factor. These findings could suggest that tolerating uncomfortable feelings is not a core part of the compassion construct. However, it would be premature to exclude the ‘tolerating’ factor from the five-element definition at this stage, given its inclusion in conceptualisations of compassion, significant factor loading, and significant intercorrelations with four of the five factors. We would therefore recommend that further empirical research explores the relationship between tolerating uncomfortable feelings and compassion more broadly and with its proposed subcomponents, the outcome of which may be that ‘tolerating’ is excluded from future definitions.

In addition to this factor-level issue, there were a number of issues with the items from existing measures loading on to the identified factors. One limitation is that not all items appear to be measuring compassion; for example, some items do not refer specifically to suffering (e.g., “I want to spend time with others so that I can find ways to help enrich their lives”) and others appeared to be measuring self-sacrificing rather than mutual compassion for self and others (e.g., “If given the opportunity, I am willing to make sacrifices in order to let other people achieve their goals in life”). Such items do not appear to be describing compassion, but instead related constructs such as kindness and altruism (see Strauss et al. [1] for a discussion of the distinction between these constructs).

Another limitation is that item wording is inconsistent across different measures (reflecting that they were derived from a range of existing measures). Additionally, only three items, which were generated through expert consultation, loaded strongly on to the ‘tolerating’ factor. Similarly, only four theoretically related items loaded strongly on to the ‘universality of suffering’ factor. This can be attributed to the paucity of items in existing self-report measures which capture these dimensions of compassion.

Limitations at the item-level such as these lead us to recommend against using these items collectively to measure compassion. A measure which includes these items is unlikely to be fully and coherently representative of compassion. Instead, we recommend the development of a new self-report questionnaire, following good practice guidelines for measure development, that is rigorously tested for its psychometric properties. Limitations at the item-level also preclude strong conclusions at this early stage regarding the conceptual structure of compassion and whether or not this should include the ‘tolerating uncomfortable feelings’ factor. Although current findings suggest that the ‘tolerating’ factor may be problematic, given its small factor loading and the lack of correlation with the ‘acting’ factor, we recommend that future research developing a new measure of compassion, which generates new items in order to overcome the item-level limitations noted above, include tolerating items in their initial item pool alongside items representing the other four elements from Strauss et al.’s [1] definition. This inclusive approach will allow future research to examine the factor structure emerging from the data, and whether tolerating is a part of this, in the absence of item-level limitations. If future research replicates current findings and highlights ‘tolerating’ as a problematic factor, then this would indicate that compassion may be better represented by four rather than five elements.

### Strengths and limitations

Strauss et al. [1] reviewed the theoretical literature on compassion and consolidated the range of conceptualisations into one multifaceted definition of compassion. Although factor-level and item-level limitations have been identified, present findings provide preliminary empirical support for a five-factor hierarchical model of compassion consistent with Strauss et al.’s [1] definition. These findings contribute to a greater understanding of the construct and provide an empirically-supported foundation for future measure development. However, the following limitations should be taken into account.

Strauss et al. [1] stated that their definition of compassion could be applied to both the self and other people. However, the current study excluded measures of self-compassion (SCS and SCS-SF) for the following reasons, also stated in the Stage 1 method section: items from the SCS [36] and SCS-SF [46] could not be easily reworded to apply to compassion towards others or compassion more generally (without specifying a target), Pommier’s [39] compassion scale and the SCS share the same factor structure (so the key elements in the SCS are represented in Pommier’s scale), and there is a lack of empirical evidence supporting a relationship between self- and other-compassion. Therefore, current findings support Strauss et al.’s [1] definition of compassion directed towards other people, but not towards the self. To advance our understanding of the conceptual structure of compassion and the relationship between self- and other-compassion, future efforts to develop a new measure of compassion based on Strauss et al.’s [1] definition should attempt to generate parallel items which can be applied to the self or others. Factor analyses could then illuminate whether the factor structure of compassion is the same when it is applied to others as it is when it is applied to the self. In addition, this approach would help to clarify the empirical relationship between self-compassion and other-compassion, as parallel items should maximise the possibility of demonstrating a relationship between the two, if one indeed exists. This line of research therefore has the potential to yield insights into the nature of the relationship and overlap between self-compassion and other-compassion.

The items in the ‘emotional connection’ and ‘tolerating uncomfortable feelings’ factors were all negatively-phrased and reverse-coded prior to EFA. Although such items were negatively-phrased, we labelled their factors positively (i.e., ‘emotional connection’ rather than ‘emotional disconnection’) so that the labels for all factors would be in the same direction and consistent in indicating compassion, rather than lack of compassion (cf. the labelling of judging items as ‘non-judging’ in the FFMQ [66]). By doing this, we are not asserting that, for example, emotional connection is the exact converse of emotional disconnection; this would need testing empirically. Our aim was simply to examine the factors emerging from exploratory factor analysis of existing compassion items. This is an initial step in the process of empirically validating the conceptual structure of compassion and providing a foundation for future measure development work. Limitations of the items which emerged from the analysis, including a reliance of negatively-phrased items for certain factors, strengthens our proposal for a new measure of compassion with newly developed items. We recommend that future research developing a new measure of compassion generate primarily positively-worded items, to reduce the possibility of factors emerging which consist solely of negatively-worded items and may not be ideally captured by a positively-phrased label.

EFA and CFA of compassion items were also limited to data from samples of University students. Using different samples (e.g., meditators, older populations, healthcare professionals) could result in greater support for the five-factor structure of compassion or the identification of alternative factor structures. However, even if further research replicated the current study using samples from more diverse populations, it is likely that similar item-level limitations will be present (e.g., poor face validity of items, inconsistent wording of items from different measures, fewer items loading on to particular factors). Therefore, we recommend that future research develop a new measure of compassion, in order to overcome the item limitations highlighted here. The factor structure of this new measure should then be tested widely in different populations.

### Future research

Findings from this study open up new avenues of research into compassion, which we are currently exploring. The primary focus of future research should be to develop a psychometrically strong self-report measure of compassion using Strauss et al.’s [1] comprehensive five-element definition to guide item generation. The development of a high quality measure which captures the key dimensions of compassion will bring us closer to being able to answer the important question of whether compassion can be cultivated through compassion-based interventions. In order to answer this important question, we first need a valid and reliable measure of compassion for the self and others in order to ascertain the effect of compassion-based interventions on each of these aspects of compassion. Such a measure may also help to optimize the effectiveness of these interventions by identifying the elements requiring greater therapeutic attention. Moreover, if this line of research supports the Buddhist perspective and demonstrates that enhancing self-compassion supports the development of compassion towards others, then compassion-based interventions may be more effective in cultivating compassion towards others if they begin by enhancing self-compassion. Conversely, if emerging research does not support a relationship between compassion towards others and self-compassion, then we cannot assume that interventions designed to cultivate one form of compassion would necessarily enhance the other form and interventions would need to clarify their focus (in terms of whether they are designed to cultivate self-compassion, compassion towards others, or both).

Although the current study supported Strauss et al.’s [1] five-element definition, a definitive answer to the question of definition is still emerging, and theoretical and empirical developments (including the development of a new scale which comprehensively captures the elements of compassion) may also shed more light on which elements are integral to compassion (and whether ‘tolerating uncomfortable feelings’ is an integral element of compassion) and how elements interact to give rise to compassion.

Future research could also explore non-self-report methods of assessing compassion (e.g., behavioural or physiological measures) and triangulate these alternative measures with a new self-report measure of compassion.

### Conclusions

The scientific study of compassion is in its infancy and many key questions remain poorly answered, including the fundamental question of measurement. As an essential first step to advancing our understanding of compassion and furthering research in this field, the current study empirically examined the conceptual structure of compassion using self-report items. Findings showed preliminary support for a five-factor structure of compassion consistent with Strauss et al.’s [1] definition; compassion consists of recognising suffering, understanding the universality of suffering in human experience, emotionally connecting with the person who is suffering, tolerating uncomfortable feelings aroused, and being motivated to act or acting to alleviate suffering. However, our findings indicated that the ‘tolerating uncomfortable feelings’ factor may be problematic and compassion may be better represented by four rather than five elements. This possibility requires further empirical testing. There were also limitations with items from included self-report measures (e.g., not all items appear to measure compassion, inconsistency in item wording across measures, lack of items representing certain elements of compassion), which lead us to recommend against using these items collectively to assess compassion. Taken together, we recommend developing a new measure of compassion, by generating self-report items for each of the five elements (including ‘tolerating’ items, to determine whether or not factor-level issues are resolved once item-level issues are addressed), and rigorously testing its psychometric properties in diverse populations.

## Supporting information

S1 File Pool of compassion items from Stage 1. (DOCX)

S2 File Stage 2 (EFA) data (*N* = 206). (SAV)

S3 File Stage 3 (CFA) data (*N* = 256). (SAV)
