# Supplementary material for: An empirical examination of the factor structure of compassion
Source: PLoS One. 2017 Feb 17;12(2):e0172471. doi: 10.1371/journal.pone.0172471 (PMC5315311; doi:10.1371/journal.pone.0172471)
Supplement: S1 File — (DOCX) [file pone.0172471.s001.docx]

Supplementary Information (S1 File) for ‘An Empirical Examination of the Factor Structure of Compassion’

**Pool of 80 Compassion Items from Stage 1**

The origin of each item is given in brackets: CLS = Compassionate Love Scale (Sprecher & Fehr, 2005); CS-M = Martins et al.’s (2013) Compassion Scale; CS-P = Pommier’s (2010) Compassion Scale; EC = generated through expert consultation; RCS = Relational Compassion Scale (Hacker, 2008).

^a^ Only item stems (50% or less of the full items) are given for items from CS-P. For full items, please refer to Pommier (2010).

1. I notice when people are upset…^a^ (CS-P).
2. If I see someone going through a difficult…^a^ (CS-P).
3. I try to put myself in other people’s shoes when they are in trouble (CLS).
4. My heart goes out…^a^ (CS-P).
5. When other people are upset, I try to be warm, sensitive and sympathetic to them (RCS).
6. I feel uncomfortable being around people who are suffering (EC).
7. If a person needs help, I would do almost anything I could to help him or her (CLS).
8. I know that everyone feels down…^a^ (CS-P).
9. I feel happy when I see that other people are happy (CLS).
10. If someone does something disagreeable I find it hard to be kind to them when they are in need (EC).
11. It is easy for me to feel the pain (and joy) experienced by other people (CLS).
12. It is important to me to recognize that…^a^ (CS-P).
13. When other people are emotionally upset I treat them with kindness and care (RCS).
14. I pay careful attention when…^a^ (CS-P).
15. When I hear about a person going through a difficult time, I feel a great deal of compassion for him or her (CLS).
16. I find it difficult to notice when people are upset (EC).
17. I am caring towards others when they are distressed (RCS).
18. I find it difficult to understand why other people get upset (EC).
19. When someone is suffering it can be hard to help them because it is so upsetting (EC).
20. Others can trust that I will be there for them if they need me (CLS).
21. When I’m busy, I do not notice other people’s emotions (EC).
22. If I am upset by another's suffering, I am able to tolerate my own emotions and not get carried away by them (EC).
23. If given the opportunity, I am willing to make sacrifices in order to let other people achieve their goals in life (CLS).
24. I think that sometimes people get upset over nothing (EC).
25. When someone is upset I help them without judging them (EC).
26. Despite my differences with others, I know…^a^ (CS-P).
27. When someone is troubled, I feel extreme tenderness and caring (CLS).
28. If someone is in distress or trouble, I wait for other people to respond first (EC).
29. I can’t really connect with…^a^ (CS-P).
30. I want to spend time with others so that I can find ways to help enrich their lives (CLS).
31. I believe that suffering is just…^a^ (CS-P).
32. When people tell me about their problems, I…^a^ (CS-P).
33. I don’t concern myself…^a^ (CS-P).
34. I show understanding and caring towards others (RCS).
35. I very much wish to be kind and good to other people (CLS).
36. I find it hard to understand other people’s problems (RCS).
37. I am moved by other people's suffering (EC).
38. I would be willing to give money to help someone in need of financial help (CS-M).
39. Thinking of other people’s suffering as part of common human experience doesn’t come easily to me (EC).
40. I like to be there…^a^ (CS-P).
41. I find it difficult to recognise how other people are feeling if they don't say anything (EC).
42. I am interested to understand others' experiences and emotions (RCS).
43. I would not be willing to help someone who I find unpleasant (EC).
44. I am attuned to other peoples' feelings (RCS).
45. When other people are emotionally upset I try to see their thoughts and feelings as valid (RCS).
46. I would be willing to share my home with someone in need who poses no threat (CS-M).
47. I feel detached from others…^a^ (CS-P).
48. When I see someone suffering I think about how I too could be in their position (EC).
49. I can understand why other people get upset (EC).
50. One of the activities that provides me with the most meaning to my life is helping others (CLS).
51. When people cry in front of…^a^ (CS-P).
52. I accept other people even when they do things I think are wrong (CLS).
53. I listen patiently when…^a^ (CS-P).
54. Even if it is upsetting hearing about someone's problems, I do my best to listen (EC).
55. When I see people feeling sad, I feel a need to reach out to them (CLS).
56. I don’t feel emotionally…^a^ (CS-P).
57. It is hard for me to relate to others when I see them suffering (EC).
58. When others are feeling troubled, I…^a^ (CS-P).
59. I feel compassion for other people (CLS).
60. When I see someone feeling upset I feel so overwhelmed by my emotions that I find it difficult to help them (EC).
61. I am accepting of people who are in need whatever their experiences (EC).
62. When others feel sadness…^a^ (CS-P).
63. I find it easy to recognise when someone is suffering or in need (EC).
64. When people talk about their problems…^a^ (CS-P).
65. I try to avoid people who…^a^ (CS-P).
66. I am empathetic towards others when they make a mistake (RCS).
67. I don't know what to do when other people are distressed (RCS).
68. I can understand how people are feeling even if I do not identify with their experiences (EC).
69. I am sensitive to the wellbeing of others (RCS).
70. I get carried away by my own emotional response to other people's problems or suffering (EC).
71. I notice when someone is different from how they usually are (EC).
72. I would be willing to give my time freely to work for someone who needs my skills but cannot afford to pay me (CS-M).
73. I try to understand rather than judge people (CLS).
74. I am cold to…^a^ (CS-P).
75. I tune out when people…^a^ (CS-P).
76. If someone is suffering I go out of my way to help them if I can (EC).
77. I have tender feelings towards others when they seem to be in need (CLS).
78. When I see someone feeling down, I…^a^ (CS-P).
79. I spend a lot of time concerned about the well-being of other people (CLS).
80. If someone is suffering I feel a natural response to want to help (EC).
